# Non‐woven bilayered biodegradable chitosan‐gelatin‐polylactide scaffold for bioengineering of tracheal epithelium

**DOI:** 10.1111/cpr.12598

**Published:** 2019-03-21

**Authors:** Olga A. Romanova, Timur H. Tenchurin, Tatiana S. Demina, Elena V. Sytina, Alexey D. Shepelev, Stanislav G. Rudyak, Olga I. Klein, Sergey V. Krasheninnikov, Elizaveta I. Safronova, Roman A. Kamyshinsky, Vissarion G. Mamagulashvili, Tatiana A. Akopova, Sergey N. Chvalun, Andrey A. Panteleyev

**Affiliations:** ^1^ Kurchatov Complex of NBICS Technologies NRC Kurchatov Institute Moscow Russian Federation; ^2^ Enikolopov Institute of Synthetic Polymeric Materials, Russian Academy of Sciences Moscow Russian Federation; ^3^ Sechenov First Moscow State Medical University Moscow Russian Federation; ^4^ Emanuel Institute of Biochemical Physics, Russian Academy of Sciences Moscow Russian Federation

**Keywords:** airway, cell differentiation, electrospinning, pseudostratified epithelium, tissue equivalent

## Abstract

**Objectives:**

The conversion of tissue engineering into a routine clinical tool cannot be achieved without a deep understanding of the interaction between cells and scaffolds during the process of tissue formation in an artificial environment. Here, we have investigated the cultivation conditions and structural features of the biodegradable non‐woven material in order to obtain a well‐differentiated human airway epithelium.

**Materials and methods:**

The bilayered scaffold was fabricated by electrospinning technology. The efficiency of the scaffold has been evaluated using MTT cell proliferation assay, histology, immunofluorescence and electron microscopy.

**Results:**

With the use of a copolymer of chitosan‐gelatin‐poly‐l‐lactide, a bilayered non‐woven scaffold was generated and characterized. The optimal structural parameters of both layers for cell proliferation and differentiation were determined. The basal airway epithelial cells differentiated into ciliary and goblet cells and formed pseudostratified epithelial layer on the surface of the scaffold. In addition, keratinocytes formed a skin equivalent when seeded on the same scaffold. A comparative analysis of growth and differentiation for both types of epithelium was performed.

**Conclusions:**

The structural parameters of nanofibres should be selected experimentally depending on polymer composition. The major challenges on the way to obtain the well‐differentiated equivalent of respiratory epithelium on non‐woven scaffold include the following: the balance between scaffold permeability and thickness, proper combination of synthetic and natural components, and culture conditions sufficient for co‐culturing of airway epithelial cells and fibroblasts. For generation of skin equivalent, the lack of diffusion is not so critical as for pseudostratified airway epithelium.

## 1 INTRODUCTION

The factual knowledge, accumulated by tissue engineering so far, is not sufficient for a transition from the empirical to a fundamentally new, rationale‐based level. Without a thorough understanding of mechanisms, that drive tissue formation in an artificial environment and the interaction of cultured cells and biodegradable scaffolds, one could not expect any significant progress in the field.

In epithelia bioengineering, biodegradable materials are of primary interest, since the restoration of the barrier function requires a timely replacement of an artificial scaffold with a native tissue, thus preventing the infection and excessive scarring.^1^ The process of formation and maturation of the functional epithelium requires reciprocal interaction of epithelial and mesenchymal cells. The epithelial cells need a thin and dense support (equivalent of a basement membrane) preventing their downward migration and promoting their flattening and “epithelialization” of the surface. By contrast, mesenchymal cells (ie, fibroblasts) require a porous substrate (equivalent of *lamina propria*) to ensure their inward migration. Thus, fabrication of a bilayered scaffold seems to be the way to solve the problem of epithelia bioengineering.

Two types of bilayered scaffolds for the 3D cultivation of respiratory epithelium deserve attention at this stage. The first type represents a bilayered matrix combining a film‐like top layer and porous sub‐layer (a collagen‐ or collagen/hyaluronate‐based sponge). Although such collagen‐based materials are sufficient in terms of biocompatibility and biodegradability, they are not providing adequate mechanical properties.^6^ Another material, proposed by Morris and coworkers,^7^ also represents a bilayered but non‐woven scaffold made from a non‐biodegradable polyethylene terephthalate. This scaffold mimics the fibrous structure of a natural extracellular matrix (ECM) of the decellularized trachea and was shown to be suitable for co‐cultivation of fibroblasts (in the microfibrous layer) and lung adenocarcinoma CALU3 cells (placed on top of a nanofibrous layer) in a bioreactor.^8^ Surprisingly, while two types of scaffold discussed above are probably the best of their kind, a solid proof that they are sufficient to generate functional equivalents of the airway epithelium is still lacking. So far no data have been reported on the developing of differentiated airway epithelium with primary human cells on non‐woven scaffolds.

Here, we obtained a novel mechanically sound polymeric scaffold that combines the advantages of both types of scaffolds mentioned above. We investigated the structural features of the biodegradable non‐woven material and the cultivation conditions to obtain a well‐differentiated human airway epithelium equivalent. On the same scaffold, we obtained skin equivalent with keratinocytes and analysed growth and differentiation for both types of epithelium. The scaffold was identified as a prospective tool for bioengineering of epithelial tissues including both airway epithelium and skin.

## 2 MATERIALS AND METHODS

### 2.1 Scaffold fabrication

#### 2.1.1 Processing of the blend

Chitosan/gelatin/poly‐l‐lactide (PLLA) blend (52:13:35 wt.%) was prepared by solid‐state reactive extrusion in a pilot ZE 40 twin‐screw extruder (Berstorff, Germany) and stabilized by fraction of chitosan‐g‐poly‐l‐lactide copolymers formed in situ in the process.

### 2.2 Scaffold fabrication

Copolymer films were cast from a stable colloidal solution of chitosan‐gelatin‐poly‐l‐lactide copolymer (CGP) in dichloromethane (DCM; Component‐Reactiv, Russia) as previously described.^10^

A bilayered fibrous matrix was obtained from CGP polymer dispersions in DCM and DCM:ethyl alcohol (Himzakaz, Russia) using electrospinning technique with equipment previously described.^11^ Addition of PLLA (4032D; NatureWorks, USA) to CGP dispersion (53% CGP + 47% PLLA) allowed to obtain a matrix with the desired mechanical properties. The parameters of a time‐stable spinning process are described in Table 1. Three types of matrices with nanolayer 25, 50 and 100 μm thick were obtained.

**Table 1:** Electrospinning parameters and dispersion composition for generation of distinct layers in CGP100‐based bilayered fibrous matrix

Layer	Solvent	Volume flow rate, mL/min	Conductivity, μS/cm	Total polymer part in the dispersion, %	Voltage, kV	Interelectrode distance, cm
Nanolayer	90% DCM 10% EA	3	0.8	17.2	22	21
Microlayer	DCM	10	0.08			

DCM, dichloromethane; EA, Ethyl alcohol.

### 2.3 Surface modification

Direct current discharge plasma modification (surface activation) was carried as described earlier.^10^ Immobilization of hyaluronic acid (HA) (MW ~5 kDa; Bloomage Freda Biopharm, China) was performed immediately after surface activation through incubation of the scaffolds in 2 wt.% aqueous solution of HA for 2 hours at room temperature. An excess of HA was washed out by bi‐distilled water and then the samples were freeze‐dried.

### 2.4 Analysis of the polymer scaffold properties

#### 2.4.1 Morphology of the obtained samples

Surface morphology of the samples was evaluated using scanning electron microscope Versa3D DualBeam (FEI, USA). The images were obtained in secondary electron mode with ultralow accelerating voltage of 1 kV.^12^ For scanning electron microscopy (SEM) image processing and assessment of the average fibre diameter, Fiji software^13^ was used. The size distribution histograms had been obtained from at least 100 fibres.

#### 2.4.2 Assessment of porosity of a bilayered fibrous matrix

For porosity (Δ%) assessment, the 4 × 4 cm scaffold samples were used. The porosity (%) of the samples was calculated from the ratio between the volume occupied by the fibres and the total volume of the sample in triplicate. The analytical value of the composite fibre density was used for calculations, under the assumption that it approaches the value of the amorphous‐crystalline PLLA density (1.2 g/cm^3^). The thickness of the scaffold layers was measured using SEM images (at least five times for each sample).

#### 2.4.3 Pore size analysis

The average pore size of the scaffolds was calculated in triplicate using POROLUX™ 1000 capillary flow porometer (Porotec, Germany) according to the manufacturer's protocol.

### 2.5 Mechanical properties of scaffold samples

The mechanical properties of matrices were determined in triplicate by Instron‐5965 Universal Testing System (Instron, USA). The size of the tested area was 10 × 5 mm, and the rate of the sample extension was 10 mm/min. The preload was 0.01 N. All studies were carried out at 23 ± 2°C. Before the experiment, all samples were contained in a liquid medium and after the test they were dried in a vacuum oven at 23°C to constant weight. To calculate the strength of the samples, the conditional cross section was determined using their mass and polymer density.

### 2.6 3D cell culture

#### 2.6.1 Isolation of primary cells

Primary cells were isolated from surgical discard tissues. Written informed consents were obtained for each volunteer and approved by Sechenov First Moscow State Medical University, Moscow, Russian Federation.

*Airway epithelial cells* were extracted from trachea. Cell isolation was performed using 0.1% Protease XIV (Sigma, Cat. No. P5147) solution as described.^14^ The extracted cell pellets were resuspended in an airway epithelial growth PneumaCult™‐Ex Medium (Stemcell Technologies, Cat. No. 05008), seeded onto collagen I (Thermo Fisher Scientific, Cat. No. A1048301)‐coated plates and cultivated in the medium with antibiotics (penicillin 100 U/mL, streptomycin 100 µg/mL, gentamicin 50 µg/mL) and antimycotic (amphotericin 0.25 µg/mL).

*Tracheal fibroblasts and dermal fibroblasts* were isolated as previously described^15^, ^16^ and grown in DMEM containing 10% foetal bovine serum (FBS). *Mesenchymal stem cells (MSCs)* were isolated as previously described^17^ from normal human gingival tissue obtained after routine dental surgery.

*Epidermal keratinocytes* were isolated from normal human skin (surgery leftovers) and grown in KSFM medium (Thermo Fisher Scientific, Cat. No. 17005042) according to the manufacturer's protocol.

The cells from three different donors were used in each experiment.

#### 2.6.2 Two‐chamber cell culture system

For the cultivation at air‐liquid interface conditions (ALI), a customized culture system was created similar to the one previously described.^5^ Polymer membranes were removed from 12‐mm‐diameter cell culture inserts (Millicell, Cat. No. PIHP01250 and Corning, Cat. No. 3460), and the matrix was clamped between their plastic frames. The construct was placed into a 12‐well plate.

#### 2.6.3 Cultivation of fibroblasts to evaluate their effects upon mechanical characteristics of the matrix

For mechanical testing, fibroblasts were seeded onto the microfibrous surface of the scaffold and cultivated as described below in Section 2.4.3 for 14 or 30 days.

#### 2.6.4 Development of complex (multicellular) equivalents

The matrix was pre‐moistened in DMEM at 37°C for 1 hour before cell seeding. Then, the medium was removed and fibroblasts were seeded onto the microfibrous surface of the material at the density of 0.25 × 10^5^ cells/cm^2^. After cultivation for 7‐14 days, epithelial cells were seeded onto the nanofibrous surface at the density of 3 × 10^5^ cells/cm^2^ and cultivated submerged for 2‐3 days in proliferative medium. Then, the proliferative medium was removed and the differentiation medium added to the bottom chamber only (ALI cultivation). The medium was changed every 2nd day.

To promote proliferation of airway epithelial cells and epidermal keratinocytes during their submerged cultivation, PneumaCult™‐Ex Medium and KSFM were used, respectively; for induction and support of cell differentiation, PneumaCult‐ALI Medium (Stemcell Technologies, Cat. No. 05001) and CnT‐Prime 3DBarrier Medium (CELLnTEC, Cat. No. CnT‐PR‐3D) were used, respectively.

Each type of equivalent was obtained at least three times with cells from different donors every time.

#### 2.6.5 Development of monoculture epithelial equivalents

The epithelial cells were seeded on pre‐soaked matrices and cultivated as described above. For positive control, 12‐mm‐diameter cell culture inserts (Millicell, Cat. No. PIHP01250) were used along with CGP matrices.

### 2.7 Assessing the properties of cell‐matrix constructs

#### 2.7.1 Cell viability analysis

Evaluation of the proliferation was carried out using the MTT cell proliferation assay (Sigma, Cat. No. M5655) as described previously (http://www.amsbio.com) on days 1, 3 and 10 after seeding 0.12 × 10^5^ cells/cm^2 ^(three replicates for each time point). The absorbance was measured using VICTOR X3 Microplate Reader (PerkinElmer, USA).

For viability analysis, fluorescent viability/cytotoxicity Kit (Invitrogen, Cat. No. L‐3224) was used according to the manufacturer's protocol*.* The samples were analysed under the fluorescence microscope Zeiss Axiovert 40 CFL.

#### 2.7.2 Histological analysis

The equivalents were fixed with 1.5% glutaraldehyde and 2% osmium tetroxide (SPI‐Chem, Cat.No. 02601‐AB), followed by dehydration through a graded series of ethanol and embedded in Epon 812 resin (SPI‐Chem, Cat. No. 02660‐AB). Semi‐thin sections (1 μm) were sliced on ultramicrotome Ultracut UC‐6 (Leica) and stained with Giemsa solution (Merck Chemicals, Cat. No. 61803900251730).

Frozen sections (12 μm) were sliced on Cryostat Microm HM 525 (Thermo Scientific). Haematoxylin & Eosin staining was carried out according to standard protocol.

#### 2.7.3 Immunofluorescence analysis

Frozen sections were stained with primary antibodies to cytokeratin 5 (Abcam, Cat. No.53121), cytokeratin 10 (Abcam, Cat. No. 76318), cytokeratin 14 (Abcam, Cat. No. 7800), collagen IV (Abcam, Cat. No. 6586), β‐catenin (Abcam, Cat. No. 16051), β‐tubulin 4 (Abcam, Cat. No. 11315) and mucin 5AC (Abcam, Cat. No. 3649) followed by staining with species‐specific secondary fluorescent antibodies (Jackson Immuno Research Cat. No. 711546152, 715165150). Finally, samples were counter‐stained with DAPI (Sigma, Cat. No.  D9542) and mounted in ImmuMount (Thermo Scientific, Cat. No. 9990402). Data were analysed using a fluorescent Zeiss Imager.D2 microscope software.

#### 2.7.4 Scanning electron microscopy

Cell‐seeded scaffolds were fixed overnight in 2.5% glutaraldehyde, followed by dehydration through a graded series of ethanol and dried on a critical point dryer HCP‐2 (Hitachi Company, Japan). Sputtering of gold on the sample was performed with IB‐3 Ion Coater (Eiko, Japan). Samples were examined under a scanning electron microscope Vega TC CamScan MV2300 (CamScan, UK).

### 2.8 Statistical analysis

The results were presented as a mean ± standard error. Statistical analysis was performed using the Student's *t* test. At *P*‐values <0.05, the results were considered as statistically significant.

## 3 RESULTS

### 3.1 Fabrication and modification of a bilayered material

**Figure 1:**
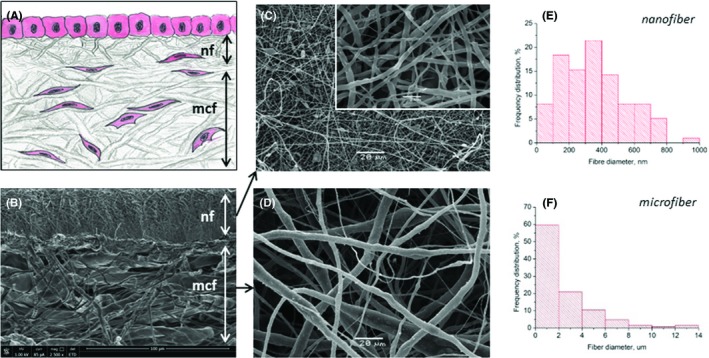
The structure of a bilayered non‐woven scaffold: (A)—a scheme of a bilayered scaffold: the microfibrous layer (mcf) was intended for the infiltration of fibroblasts. A nanofibrous layer (nf) was designed to support growth of epithelium on its surface; (B‐D)—SEM images of electrospun bilayered CGP scaffold (w/o cells): (B)—scaffold cross section showing relative thickness of the layers and their tight bonding, (C)—nanofibrous layer and the structure of its fibres (insert), (D)—microfibrous layer and the structure of its fibres (same magnification as on C); (E‐F)—histograms showing distribution of scaffold fibre diameters (measurement of at least 100 individual fibres) for nanofibrous layer (E) and microfibrous layer (F)

The morphology and structural characteristics of the obtained scaffolds are shown on Figure 1 and Table 2, respectively. Both nano‐ and microfibrous layers were homogenous, free of beads and possessed an anticipated structure. The upper (nanofibrous) layer (Figure 1A) was designed to support the growth of epithelial cells and to prevent their migration into interior space of the scaffold. The inter‐fibre distance (pore size) in this layer was 2.8 ± 0.1 μm, which corresponded to a fibre diameter of 0.35 ± 0.2 μm (Figure 1C,E and Table 2). The lower microfibrous layer was designed to allow the fibroblasts to migrate inside and evenly populate the entire volume of the layer. This layer had an inter‐fibre distance of 37.5 ± 3.8 μm, which corresponded to a fibre diameter of 2.5 ± 2.4 μm (Figure 1D,F and Table 2). The thickness of microfibrous layer was always the same (180 ± 3 μm), while for nanolayer, we tested three different options: 25, 53 and 100 μm. Both layers were tightly and securely connected to each other (Figure 1B).

**Table 2:** The characteristics of non‐woven bilayered scaffold used for bioengineering of respiratory epithelium

Parameters	Nanofibrous layer	Microfibrous layer
Thickness (μm±; SEM)	53 ± 5	180 ± 3
Porosity (%)	90 ± 3	88 ± 3
Pore size (μm±; SEM)	2.8 ± 0.1	37.5 ± 3.8
Fibre diameter (μm±; SEM)	0.35 ± 0.2	2.5 ± 2.4

After the electrospinning, the scaffold surface was hydrophobic (the water contact angle was 114°). To increase hydrophilicity of the material, the scaffold was subjected to modification comprising plasma treatment and subsequent HA immobilization. HA treatment made the measurement of the water contact angle impossible since the water was instantly absorbed into the material (Video S1).

### 3.2 Proliferation of epithelial cells depends on nanofibrous surface structure at submerged culture

**Figure 2:**
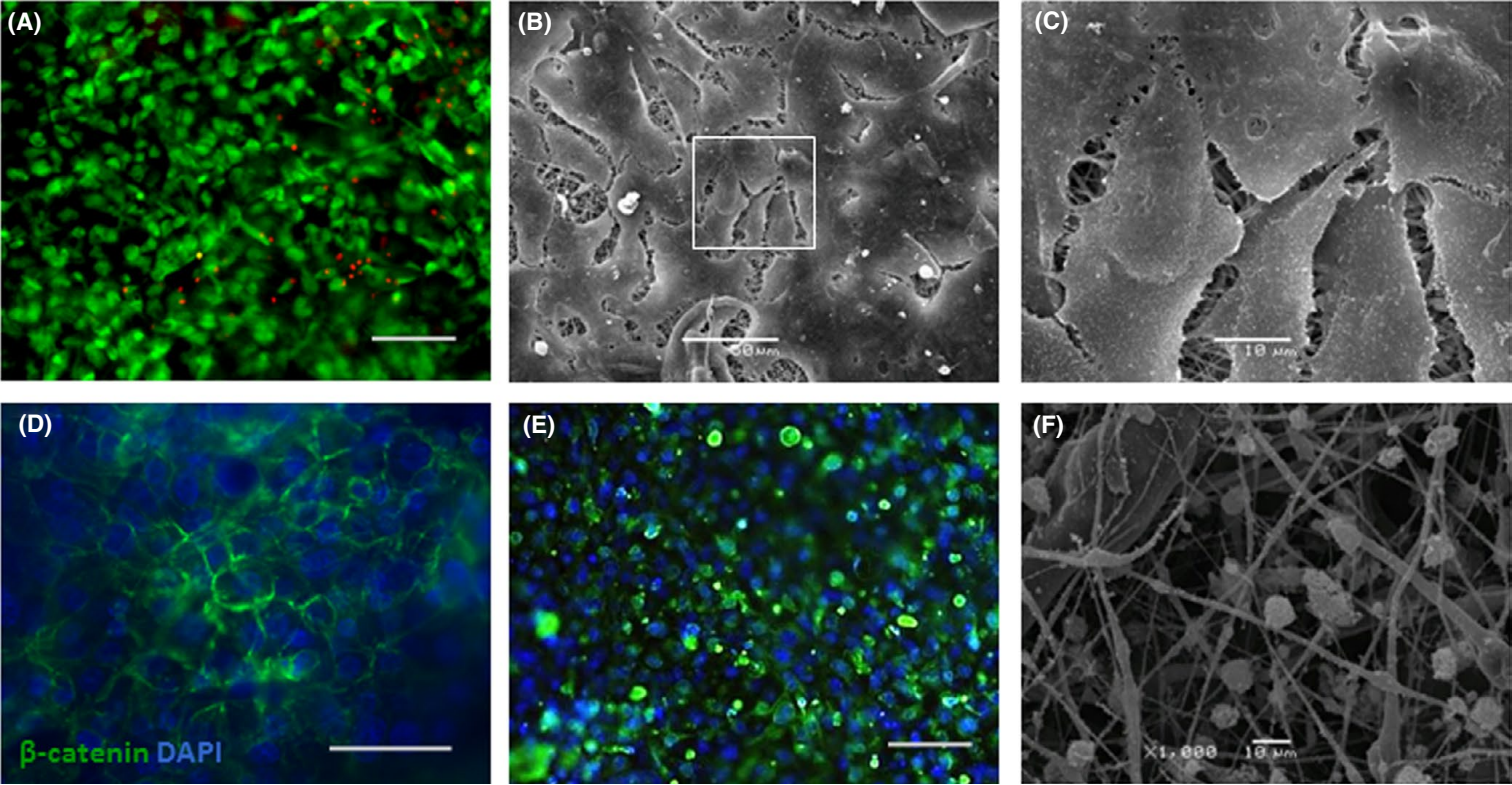
Basal cells of airway epithelium on the CGP nanofibrous layer of optimal thickness (about 50 μm), for day 10th after seeding: (A)—fluorescent viability/cytotoxicity kit showed that material is not toxic, live cells (green) cover its entire surface, dead cells are scarce (red nuclei); (B)—epithelial cells on the surface of the matrix nanolayer (SEM); (C)—higher magnification of framed zone on 4B; (D)—IF staining of epithelial cells for β‐catenin shows that CGP matrix supports formation of intercellular contacts; (E)—CGP matrix supports proliferation of the basal cells as evident from positive IF staining for CK5; (F)—SEM image of the epithelial cells (all cells are dead) on the surface of the nanolayer with inappropriate structure (pore size more than 10 μm). Scale bars: A, D, F—100 µm, B—50 µm, C, F—10µm

The fluorescence microscopy and SEM have shown that the primary human respiratory epithelium cells actively proliferate, migrate and reach confluence on the surface of the nanofibrous layer with practically no dead cells as confirmed by viability/cytotoxicity assay (Figure 2A). On days 2‐4 of culturing, the cells covered the entire surface of the matrix and formed multiple intercellular contacts, as indicated by staining with antibodies to claudin I (data not shown) and β‐catenin (Figure 2D), as well as by SEM results (Figure 2B,C). Most of the cells expressed cytokeratin 5 (CK5)—a marker of basal epithelial cells (Figure 2E). This indicates that cells retain the proliferative potential during the cultivation on the matrix and confirms their epithelial origin. Interestingly, if the pore size in the nanolayer was increased to more than 5 μm, the cells tended to form clusters but not a monolayer. On nanolayer with pores >10 μm, the epithelial cells ceased proliferation and spreading and eventually died (Figure 2F). Uneven structure of nanofibres (ie, presence of beads) also hampered monolayer formation.

**Figure 3:**
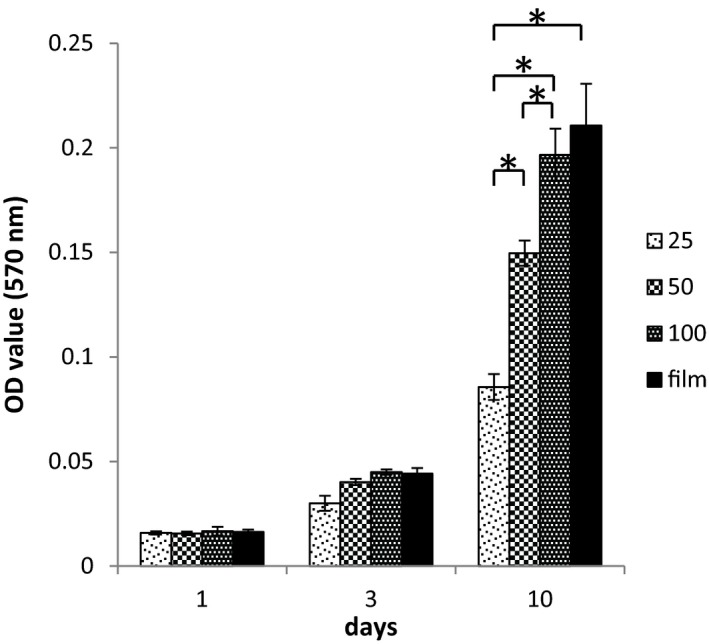
Primary airway epithelium viability depends on CGP scaffold structure: MTT cell proliferation assay results (OD value) for basal cells of human airway epithelium on CGP film or 25, 50 and 100 µm thick nanofibrous layer (1, 3 and 10 days in culture). *P*‐value <0.05 is indicated by asterisk (*) for day 10th

The thickness of the nanolayer also had a prominent effect on the proliferation and migration of epithelial cells (Figure 3). To reach a compromise between the rates of culture medium diffusion through the nanolayer during ALI cultivation and ability of this layer to provide adequate support for epithelialization, we have selected for further experiments the nanolayer of medium thickness of 53 ± 5 μm (Figure 1B and Table 2). The nanolayer of 25μm thickness appeared to be too rarefied to support cell migration, while 100 μm layer was too thick to provide sufficient nutrient diffusion to the air‐exposed cells on its surface.

### 3.3 Development of a differentiated airway epithelium on bilayered scaffold at ALI

#### 3.3.1 Differentiation of epithelial cells at ALI

**Figure 4:**
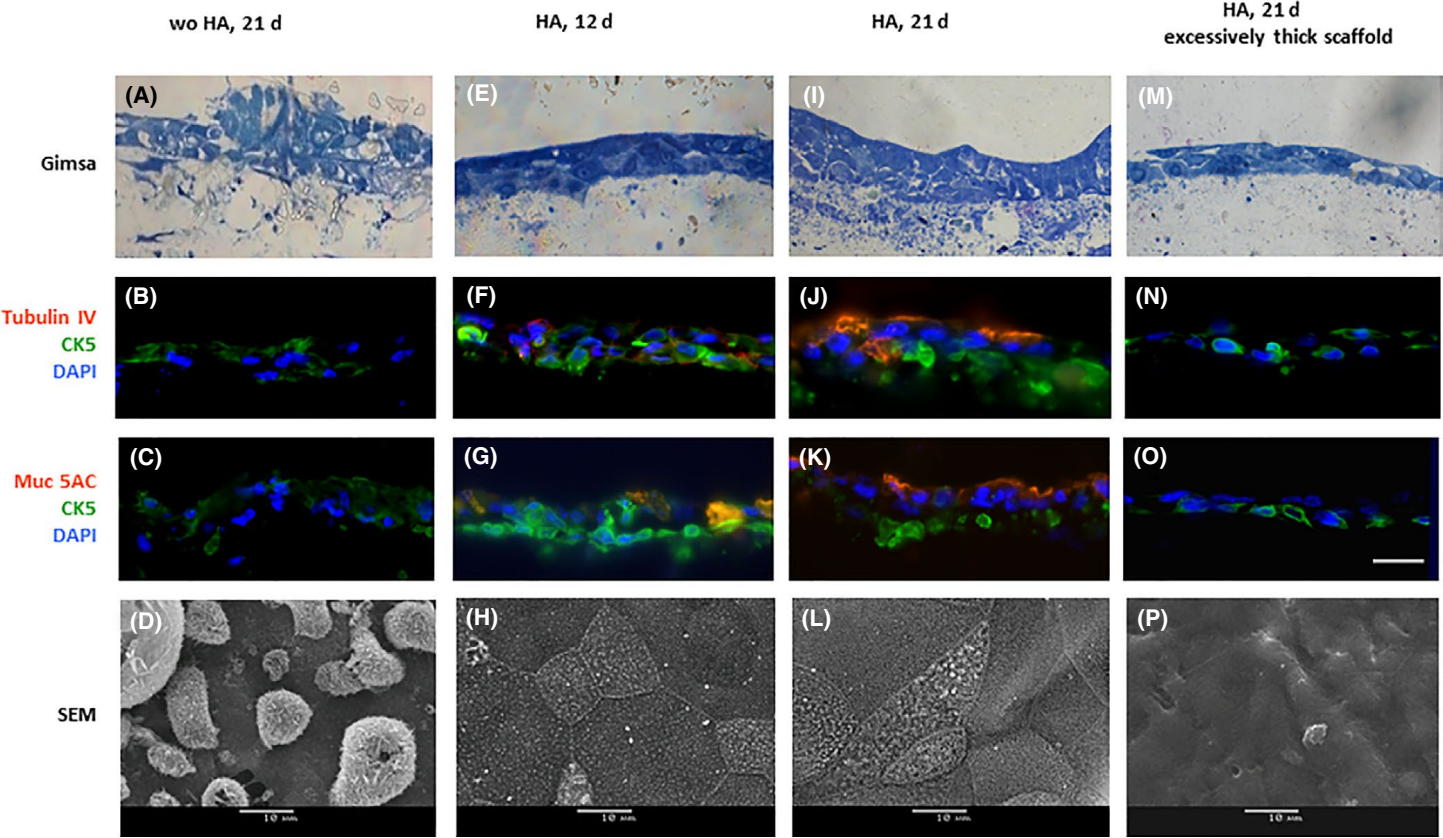
3D culture of airway epithelium cells on the bilayered scaffolds: (A‐D)—scaffold without HA modification on the 21th day of culture at ALI: cells were proliferating, but the pseudostratified epithelium did not form (no Muc5AC and Tubulin IV positivity, no ciliated cells); (E‐H)—scaffold with immobilized HA on the 12th day of cultivation at ALI: “pseudo‐layers” are visible (E), weak expression of tubulin IV (F) and pronounced expression of Muc5AC (G) are detected; the epithelium is covered with evenly distributed short cilia (H); (I‐L)—scaffold with immobilized HA on the 21st day of ALI culture: the pseudostratified epithelium (I) with dystrophic basal layer and cilia (L) is seen; the expression of tubulin IV (J) and mucin 5AC (K) is clearly detected; (M‐P)—excessively thick scaffold with immobilized HA on the 21th day of differentiation: failure of mucociliary differentiation and formation of squamous epithelium (M), the expression of tubulin IV (N) and mucin 5AC (O) is not detected, cilia are not formed (P). A, E, I, M—semi‐thin sections (Giemsa staining); B, F, J, N—IF staining for tubulin IV and CK 5; C, G, K, O—IF staining for mucin 5AC and CK 5; scale bar 50 µm. D, H, L, P—SEM images of the surface of CGP scaffold, scale bars 10 µm

When cultivated on a non‐modified matrix (total thickness of 235 µm, no HA treatment), the epithelial cells survived and proliferated, but the respiratory epithelium was not formed (Figure 4A‐D).

In contrast, additional modification of the same (235 µm thick) matrix with the HA was sufficient to ignite and support epithelial cell differentiation with eventual formation of apparently normal airway epithelium. On the 12th day at ALI, weak expression of ciliary marker tubulin IV and goblet cells marker mucin 5AC was detected (Figure 4F,G). On the 21st day, the ciliary and goblet cells maturation continued (Figure 4I‐L), as indicated by the noticeable expression of tubulin IV and mucin 5AC (Figure 4J,K). The presence of ciliated cells was also confirmed by the SEM results (Figure 4H,L). However, the basal cells pool was depleted (Figure 4M‐P) as evident from the nuclei fragmentation in basal CK5‐positive cells.

Noteworthy, the increase in total scaffold thickness to 350 µm resulted in suppression of mucociliary differentiation and formation of squamous epithelium irrespectively of HA treatment (Figure 4M‐P). SEM analysis revealed undifferentiated cells only with indistinct intercellular borders (Figure 4P).

### 3.4 Positive control (cell cultivation on polycarbonate inserts)

We used commercial polycarbonate inserts coated with collagen IV as a positive control model. The formation of pseudostratified epithelium was confirmed by microscopy (Figure S1).

### 3.5 Co‐cultivation of epitheliocytes with fibroblasts did not lead to increase in cell differentiation rates

The microfibrous layer retained mechanical properties (Figure S2) and supported the growth of tracheal fibroblasts and gingival MSCs (Figure S3). However, neither of these cell types was capable of stimulating epithelial cell differentiation upon co‐cultivation. Furthermore, in some 3D cultivation experiments, CK5 positivity was observed all over the epithelium, suggesting stimulation of proliferative response in basal cells and suppression of differentiation (Figure S3). Surprisingly, in some cases, epithelial cells even died.

### 3.6 The formation of skin equivalents on bilayered scaffold at ALI

We assumed that the combination of high biocompatibility of CGP‐based scaffold with its bilayered structure would support the formation of not only respiratory, but other types of epithelia as well. We evaluated the ability of our material to support growth of 3D skin equivalents using both primary human keratinocytes and dermal fibroblasts.

**Figure 5:**
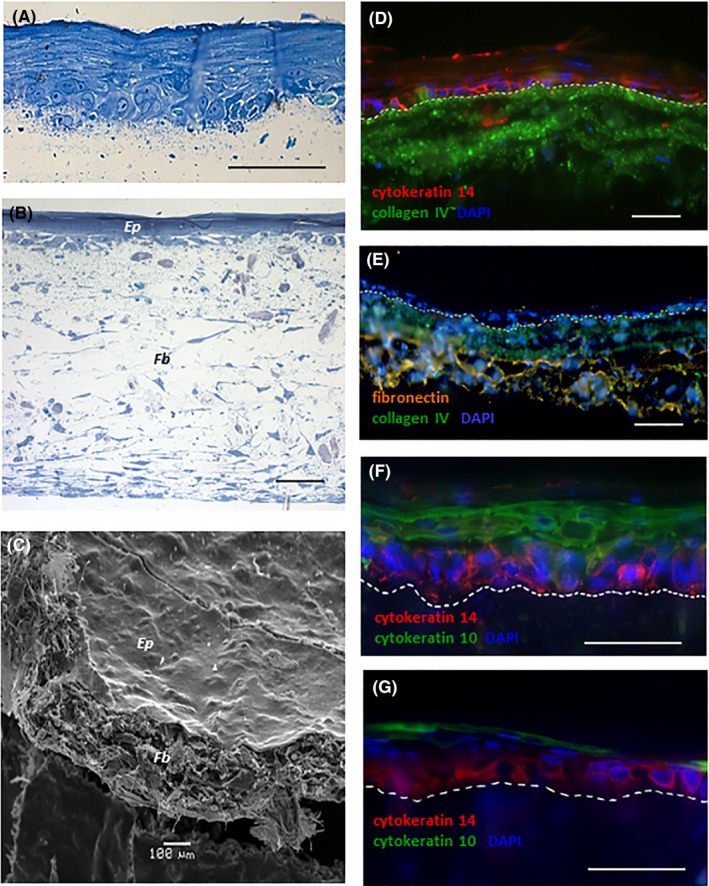
Skin equivalent on the bilayered CGP scaffold on the 9th day of ALI culture: (A)—stratified epithelium on the nanofibrous surface of the scaffold, Giemsa staining, semi‐thin section; (B)—the microfiber layer populated by dermal fibroblasts (*Fb*) and stratified epithelium (*Ep*) on the nanofibrous surface of the scaffold, Giemsa staining, semi‐thin section; (C)—skin equivalent on a bilayered scaffold: the epithelial sheet (*Ep*) on the nanofibrous surface with dermal fibroblasts (*Fb*) in the microfiber layer (SEM); (D‐G)—IF staining, frozen sections: (D)—cytokeratin 14 positivity in the basal epithelial layer and collagen IV right beneath; (E)—components of natural ECM: collagen IV (just beneath the epithelial layer) and fibronectin (synthesized by fibroblasts); (F)—cytokeratin 14 positivity in the basal epithelial layer and keratin 10 in the suprabasal cells; (G)—cell‐depleted epithelium in the centre of the scaffold without HA: expression of CK14 (basal layer) and CK10 (suprabasal layer) is decreased. The dashed line indicates the epithelium‐matrix junction. Scale bars: 50 µm, at SEM image 100 µm

The microfibrous layer supported growth and migration of dermal fibroblasts into the scaffold (Figure 5B,C). Fibroblasts synthesized fibronectin—component of natural ECM (Figure 5D). The upper nanofibrous layer supported proliferation of primary human keratinocytes. After induction of keratinocyte differentiation, epithelial equivalent was well stratified with defined basal layer (Figure 5A). Immunofluorescent staining (IF) showed expression of cytokeratin 14 in basal and cytokeratin 10 in suprabasal layers (Figure 5D,F). Keratinocytes of the basal layer synthesized the component of the basement membrane, collagen IV (Figure 5D,E). However, analysis of the morphology showed that epithelium was unevenly distributed on the matrix surface: the epithelial strata were thinner towards the centre (Figure 5F,G). Immobilization of HA resulted in more uniform epithelium morphology in the centre of skin equivalent.

As a positive control, commercial polycarbonate inserts were used (Figure S4).

## 4 DISCUSSION

### 4.1 Bilayered CGP scaffold development

The generation of a polymer scaffold for tracheal epithelium bioengineering was associated with tackling a number of technical problems which were tightly interconnected and required resolution all at the same time. Among advantages of non‐woven materials are the similarity of their structure to the natural ECM and sufficient mechanical properties. However, synthetic polymers (suitable for electrospinning) do not have natural components supporting cell‐matrix interaction, are usually highly hydrophobic and possess low biocompatibility (Table 3).

**Table 3:**
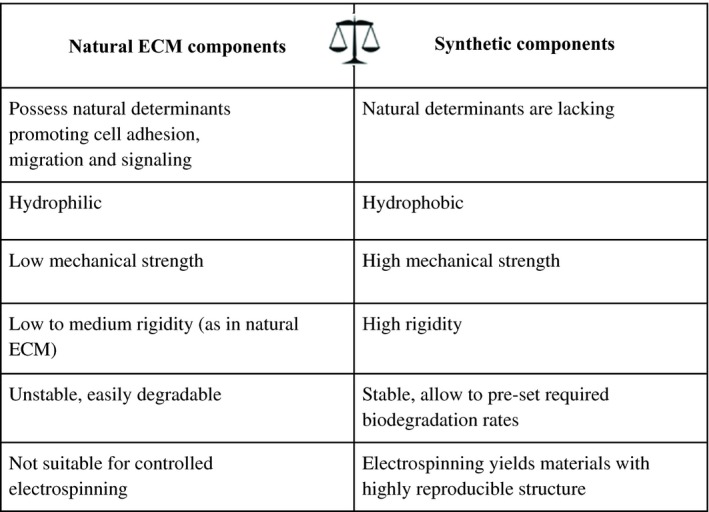
The features of synthetic polymers and natural ECM components which may affect the quality of resulting non‐woven scaffolds

Currently, cultivation of respiratory epithelial equivalents is usually performed on commercial polycarbonate membranes^19^ or on scaffolds made of collagen^20^ or of its mixtures with natural ECM components. However, the use of collagen‐based scaffolds is hampered by the insufficient mechanical properties of these materials.^6^ In addition, while being nearly ideal for cell growth support, they have poor suitability for electrospinning and excessively high biodegradation rates.^23^

To tackle these conflicting demands, we have chosen the CGP copolymer combining high mechanical properties and suitability for electrospinning of polylactide with biocompatibility, high cell adhesion capacity and antibacterial properties of chitosan.^25^ The inclusion of gelatin into CGP backbone provides natural determinants.^26^ The combination of acidic (PLLA) and basic (chitosan) polymers in one macromolecule results in minimal pH shifts during biodegradation of the material.

### 4.2 Proliferation of epithelial cells depends on nanofibrous surface structure at submerged culture

According to our results, in bilayered scaffold, the nanolayer should have small pores, be homogeneous and dense (with tightly packed fibres). The epithelial cells do not survive on a nanolayer with irregular structure and multiple beads on the fibres. The appearance of beads was directly related to the ratio of the hydrophobic to hydrophilic components in the polymer dispersion. Supplementation of the final blend with PLLA allowed to obtain more homogeneous fibres and to minimize formation of beads in the nanolayer.

The nanolayer pore size up to 5 μm appeared to be optimal for the epithelial basal cell survival and preservation of their proliferative and migratory potential for sufficient period of time (Figure 2E). When a pore size was more than 5 μm, cells formed clusters and did not survive after transfer to ALI.

The nanolayer thickness appeared to be of particular importance for the epithelial cell survival on top of the scaffold. In the submerged culture, 100 μm thick nanolayer supported cell migration and formation of a cell sheath much better as compared to 50 and, especially, 25 μm thick layers. However, during ALI cultivation, when the importance of nutrient supply from the below came to the first place, the 100 μm nanolayer was too thick to provide sufficient diffusion. Thus, for epithelial cell monolayer formation at ALI, the nanofibrous layer of the intermediate thickness (about 50 μm) appears to be optimal.

### 4.3 Development of a differentiated airway epithelium on bilayered scaffold at ALI

#### 4.3.1 Modification of CGP scaffold with hyaluronic acid

The CGP copolymer contained only a part of chitosan and gelatin, and thus cannot be designated as “natural.” To gain better control over cell differentiation on CGP matrices, they were modified with HA, that is an important structural and signalling component of natural ECM, interacting with cells through the CD44 receptor.^27^ HA treatment had also increased matrix hydrophilicity: the absorbed macromolecules of HA are guiding aqueous media components.^28^ Indeed, HA immobilization was sufficient to induce differentiation of human airway epithelium cells on nanolayer surface (Figure 4).

#### 4.3.2 The role of matrix thickness and permeability

During ALI cultivation, respiratory epithelium cells appeared to be highly dependent upon nutrient and liquid supply from the bottom. High nutrient demand of developing respiratory epithelium may be linked to high energy consumption by mucociliary transport. To fulfil these requirements, the cultivation of respiratory epithelium equivalents in perfusion bioreactors may represent a good option. It was previously reported that for artificial scaffolds thicker than 500 μm, forceful perfusion is mandatory.^29^ The perfusion leads to a measurable increase in the proliferation rate of oral mucosa cells on sponges.^30^ Of note, on thick scaffolds (over 300 µm), we did not see formation of mucociliary pseudostratified epithelium even after HA treatment. The differentiation slipped towards stratified squamous epithelium (Figure 4M‐P). Previously, it was shown that the differentiation of the tracheal epithelium depends on the porosity of the scaffold used. Our results are in line with these reports.

### 4.4 Co‐cultivation of airway epithelial cells with fibroblasts

The differentiation of the respiratory epithelium is a complex process controlled by multiple factors,^33^ which significantly vary between different mammalian species.^32^ Many of these factors are directly secreted by resident fibroblasts or are under fibroblast expressional control.^33^ The stimulation of rat and guinea pig tracheal epithelial cells differentiation by fibroblasts (with basement membrane formation) was shown in 3D culture on collagen gels. Later, it was shown that rat MSCs may affect epithelial cell activity in different ways: gingival MSCs promoted differentiation of basal tracheal epithelial cells, while adipose‐derived MSCs stimulated their proliferation.^35^ Nevertheless, the data on the role of fibroblast‐secreted paracrine factors in maintaining the balance between proliferation and differentiation of human epithelial cells are scarce.

In our experiments, we failed to achieve an effective balance between proliferation and differentiation of epithelial cells by their co‐culture with fibroblasts. The presence of fibroblasts resulted either in death of epithelial cells or in their excessive proliferation demarcated by increase in CK5 expression. Further detailed studies are necessary to fill up the gap in our understanding of epithelial/mesenchymal interactions and the formation of basal membrane, which is essential for the functioning of any epithelial tissue.^36^

### 4.5 Skin equivalent on CGP scaffold

Generation of morphologically sound skin equivalents containing two (keratinocytes and mesenchymal cells) and even three (including melanocytes) cell types has been described in sufficient details, for example, Powell and Boyce.^26^ However, a combined cultivation of respiratory epithelium cells with mesenchymal cells still remains a challenge. Our results did not make an exception. The generation of a complex equivalent of tracheal epithelium on apparently optimal CGP scaffold appeared problematic. At the same time, using the same bilayered matrix, we obtained complex skin equivalent (Figure 5A) with both keratinocytes and fibroblasts functioning in concert (Figure 5E,F).

When cultured at ALI, the epidermal keratinocytes appeared to be less sensitive to scaffold thickness and porosity as compared to respiratory epithelial cells. With increasing scaffold thickness, the differentiation of airway epithelial cells first shifted towards the flattening squamous type, and then (on thicker matrices) the cells died. In contrast, the epidermal keratinocytes survived on excessively thick (>500 μm) scaffolds and formed stratified layers but the basal layer showed signs of dystrophy. These diversities may be linked to differences in nutrient and moisturization demand between ciliated mucous‐producing respiratory epithelium and cornifying epidermis.

Furthermore, for airway epithelium, modification of the CGP scaffold by HA was mandatory to induce formation of columnar epithelium, while in skin equivalents, HA was not essential for differentiation.

## 5 CONCLUSIONS

1. Non‐woven materials appear to be extremely attractive for the production of complex epithelial equivalents. However, the production of high‐quality non‐woven scaffolds requires the use of a high proportion of synthetic components. This makes it difficult to develop mucous airway epithelial equivalents at such non‐physiological conditions.
2. The structural parameters of nanofibres should be selected experimentally depending on polymer composition and changes in surface topography due to scaffold biodegradation. For CGP copolymer used in our studies, optimal thickness of nanofibrous layer is 50 µm (less was too rarefied but more was not enough for diffusion) with pore size not more than 5 µm.
3. For a stimulating effect of fibroblasts on mucociliary differentiation of epithelial cells, proper cultivation conditions and correct mesenchymal cell source selection are of primary importance.
4. For the generation of skin equivalent, the lack of diffusion is not as critical as for pseudostratified airway epithelium. This finding may explain why attempts to create skin equivalents on different matrices are much more successful as compared with airway epithelium. The use of forceful perfusion (a bioreactor) may solve this problem.

## CONFLICT OF INTEREST

The authors declare that they have no conflict of interest.

## AUTHOR CONTRIBUTIONS

OAR conceived and designed the study. TSD and THT selected and developed the polymer material. OAR, THT, OIK, ADS, SVK, RAK, EIS and VGM collected and analysed the data. EVS and OAR drafted the manuscript. SGR and EVS statistically analysed and reviewed the manuscript. AAP acquired the funding, reviewed and edited the manuscript. TAA, SNC and AAP approved the final version of the manuscript.

## References

[1] Grillo HC . Tracheal replacement: a critical review. Ann Thorac Surg. 2002;73:1995‐2004. 12078821 10.1016/s0003-4975(02)03564-6

[2] Shoji S , Rickard KA , Ert RF , Linder J , Rennard SI . Lung fibroblasts produce chemotactic factors for bronchial epithelial cells. Am J Physiol. 1989;257(2 Pt 1):L71‐L79. 2764118 10.1152/ajplung.1989.257.2.L71

[3] Sacco O , Silvestri M , Sabatini F , Sale R , Defilippi AC , Rossi GA . Epithelial cells and fibroblasts: structural repair and remodelling in the airways. Paediatr Respir Rev. 2004;5(Suppl A);S35‐S40. 14980241 10.1016/s1526-0542(04)90008-5

[4] Tada Y , Suzuki T , Takezawa T , et al. Regeneration of tracheal epithelium utilizing a novel bipotential collagen scaffold. Ann Otol Rhinol Laryngol. 2008;117:359‐365. 18564533 10.1177/000348940811700506

[5] O'Leary C , Cavanagh B , Unger RE , et al. The development of a tissue‐engineered tracheobronchial epithelial model using a bilayered collagen‐hyaluronate scaffold. Biomaterials. 2016;85:111‐127. 26871888 10.1016/j.biomaterials.2016.01.065

[6] Brown RA . In the beginning there were soft collagen‐cell gels: towards better 3D connective tissue models? Exp Cell Res. 2013;319:2460‐2469. 23856376 10.1016/j.yexcr.2013.07.001

[7] Morris GE , Bridge JC , Brace LA , et al. A novel electrospun biphasic scaffold provides optimal three‐dimensional topography for in vitro co‐culture of airway epithelial and fibroblast cells. Biofabrication. 2014;6(3):035014. 24925127 10.1088/1758-5082/6/3/035014

[8] Bridge JC , Aylott JW , Brightling CE , et al. Adapting the electrospinning process to provide three unique environments for a tri‐layered in vitro model of the airway wall. J Vis Exp. 2015;101:e52986. 10.3791/52986 PMC4544510 26275100

[9] Akopova TA , Demina TS , Shchegolikhin AN , et al. A novel approach to design chitosan‐polyester materials for biomedical applications. Int J Polym Sci. 2012;2012:1‐10.

[10] Demina T , Zaytseva‐Zotova D , Yablokov M , et al. A DC discharge plasma modification of chitosan/gelatin/PLLA films: surface properties, chemical structure and cell affinity. Surf Coat Technol. 2012;207:508‐516.

[11] Tenchurin TK , Krasheninnikov Sn , Orekhov As , et al. Rheological features of fiber spinning from polyacrylonitrile solutions in an electric field. Structure and Properties. Fiber Chemistry. 2014;46:151‐160.

[12] Orekhov AS , Klechkovskaya VV , Kononova SV . Low‐voltage scanning electron microscopy of multilayer polymer systems. Crystallogr Rep. 2017;62:710‐715.

[13] Schindelin J , Arganda‐Carreras I , Frise E , et al. Fiji: an open‐source platform for biological‐image analysis. Nat Methods. 2012;9:676‐682. 22743772 10.1038/nmeth.2019 PMC3855844

[14] Fulcher ML , Gabriel S , Burns KA , Yankaskas JR , Randell SH . Well‐differentiated human airway epithelial cell cultures. Methods Mol Med. 2005;107:183‐206. 15492373 10.1385/1-59259-861-7:183

[15] Goto Y , Noguchi Y , Nomura A , et al. In vitro reconstitution of the tracheal epithelium. Am J Respir Cell Mol Biol. 1999;20:312‐318. 9922223 10.1165/ajrcmb.20.2.3062

[16] Takashima A . Establishment of fibroblast cultures. Curr Protoc Cell Biol. 2001;00(1):2.1.1‐2.1.2. 10.1002/0471143030.cb0201s00 18228346

[17] Zhang Q , Shi S , Liu Y , et al. Mesenchymal stem cells derived from human gingiva are capable of immunomodulatory functions and ameliorate inflammation‐related tissue destruction in experimental colitis. J Immunol. 2009;183:7787‐7798. 19923445 10.4049/jimmunol.0902318 PMC2881945

[18] Schindler M , Ahmed I , Kamal J , et al. A synthetic nanofibrillar matrix promotes in vivo‐like organization and morphogenesis for cells in culture. Biomaterials. 2005;26:5624‐5631. 15878367 10.1016/j.biomaterials.2005.02.014

[19] Crespin S , Bacchetta M , Huang S , Dudez T , Wiszniewski L , Chanson M . Approaches to study differentiation and repair of human airway epithelial cells. Methods Mol Biol. 2011;742:173‐185. 21547732 10.1007/978-1-61779-120-8_10

[20] Gray TE , Guzman K , Davis CW , Abdullah LH , Nettesheim P . Mucociliary differentiation of serially passaged normal human tracheobronchial epithelial cells. Am J Respir Cell Mol Biol. 1996;14:104‐112. 8534481 10.1165/ajrcmb.14.1.8534481

[21] O’Leary C , O’Brien FJ , Cryan SA . Retinoic acid‐loaded collagen‐hyaluronate scaffolds: a bioactive material for respiratory tissue regeneration. ACS Biomater Sci Eng. 2017;3:1381‐1393. 10.1021/acsbiomaterials.6b00561 33429696

[22] Cornelissen CG , Dietrich M , Krüger S , Spillner J , Schmitz‐Rode T , Jockenhoevel S . Fibrin gel as alternative scaffold for respiratory tissue engineering. Ann Biomed Eng. 2012;40:679‐687. 22009317 10.1007/s10439-011-0437-8

[23] Nakada A , Shigeno K , Sato T , et al. Manufacture of a weakly denatured collagen fiber scaffold with excellent biocompatibility and space maintenance ability. Biomed Mater. 2013;8:045010. 23804650 10.1088/1748-6041/8/4/045010

[24] Akopova TA , Demina TS , Zelenetskii AN . Amphiphilic systems based on polysaccharides produced by solid‐phase synthesis. Fibre Chem. 2012;44(4):217‐220.

[25] No HK , Park NY , Lee SH , Meyers SP . Antibacterial activity of chitosans and chitosan oligomers with different molecular weights. Int J Food Microbiol. 2002;74:65‐72. 11929171 10.1016/s0168-1605(01)00717-6

[26] Powell HM , Boyce ST . Fiber density of electrospun gelatin scaffolds regulates morphogenesis of dermal‐epidermal skin substitutes. J Biomed Mater Res A. 2008;84:1078‐1086. 17685398 10.1002/jbm.a.31498

[27] Ponta H , Sherman L , Herrlich PA . CD44: from adhesion molecules to signalling regulators. Nat Rev Mol Cell Biol. 2003;1:33‐45. 10.1038/nrm1004 12511867

[28] Kudryavtseva V , Stankevich K , Gudima A , et al. Atmospheric pressure plasma assisted immobilization of hyaluronic acid on tissue engineering PLA‐based scaffolds and its effect on primary human macrophages. Mater Des. 2017;127:261‐271.

[29] Kalyanaraman B , Supp DM , Boyce ST . Medium flow rate regulates viability and barrier function of engineered skin substitutes in perfusion culture. Tissue Eng Part A. 2008;14:583‐593. 18399733 10.1089/tea.2007.0237

[30] Navarro FA , Mizuno S , Huertas JC , Glowacki J , Orgill DP . Perfusion of medium improves growth of human oral neomucosal tissue constructs. Wound Repair Regen. 2001;9:507‐512. 11896993 10.1046/j.1524-475x.2001.00507.x

[31] Widdicombe JH , Sachs LA , Finkbeiner WE . Effects of growth surface on differentiation of cultures of human tracheal epithelium. Vitro Cell Dev Biol Anim. 2003;39:51‐55. 10.1290/1543-706X(2003)039<0051:EOGSOD>2.0.CO;2 12892527

[32] Cozens D , Grahame E , Sutherland E , Taylor G , Berry CC , Davies RL . Development and optimization of a differentiated airway epithelial cell model of the bovine respiratory tract. Sci Rep. 2018;8:853. 29339818 10.1038/s41598-017-19079-y PMC5770467

[33] Rock JR , Randell SH , Hogan BL . Airway basal stem cells: a perspective on their roles in epithelial homeostasis and remodeling. Dis Model Mech. 2010;3:545‐556. 20699479 10.1242/dmm.006031 PMC2931533

[34] Kobayashi K , Nomoto Y , Suzuki T , et al. Effect of fibroblasts on tracheal epithelial regeneration in vitro. Tissue Eng. 2006;9:2619‐2628. 10.1089/ten.2006.12.2619 16995795

[35] Kobayashi K , Suzuki T , Nomoto Y , et al. A tissue‐engineered trachea derived from a framed collagen scaffold, gingival fibroblasts and adipose‐derived stem cells. Biomaterials. 2010;18:4855‐4863. 10.1016/j.biomaterials.2010.02.027 20347137

[36] Gumbiner BM . Cell adhesion: the molecular basis of tissue architecture and morphogenesis. Cell. 1996;84:345‐357. 8608588 10.1016/s0092-8674(00)81279-9

