# Supplementary figures and images for: Non‐woven bilayered biodegradable chitosan‐gelatin‐polylactide scaffold for bioengineering of tracheal epithelium
Source: Cell Prolif. 2019 Mar 21;52(3):e12598. doi: 10.1111/cpr.12598 (PMC6536443; doi:10.1111/cpr.12598)

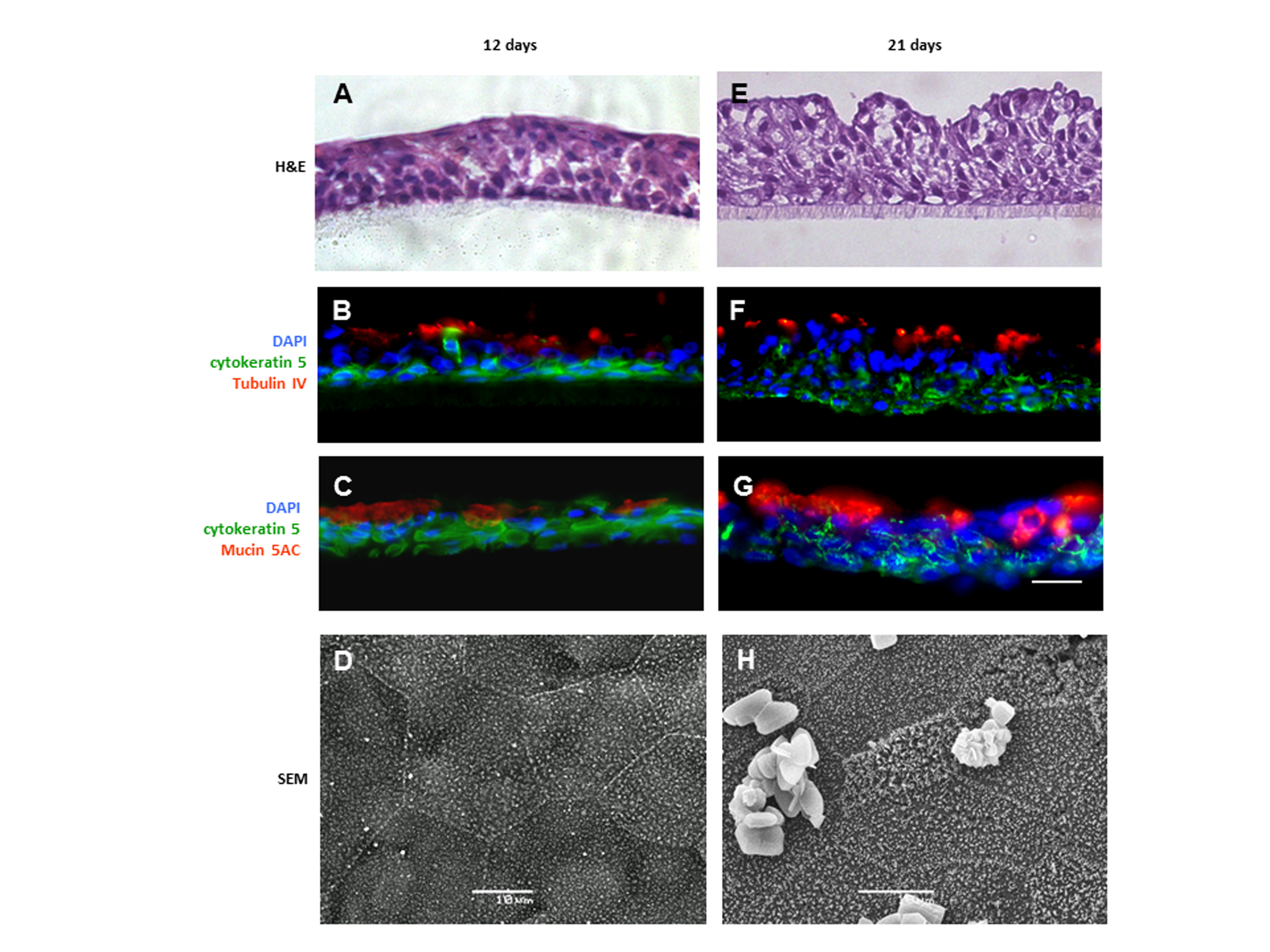

Supplement: Supplementary file 1 [file CPR-52-e12598-s001.tif]

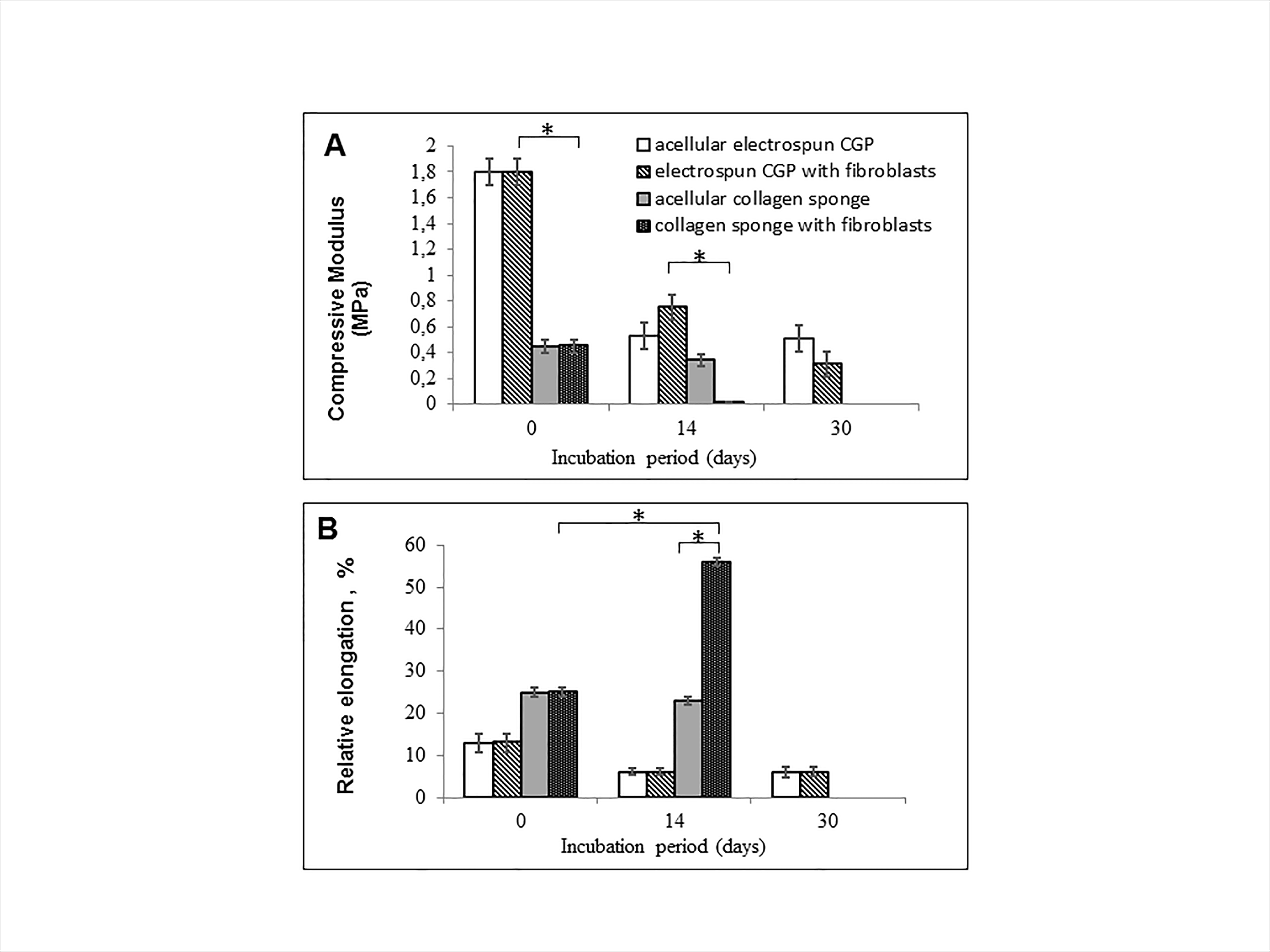

Supplement: Supplementary file 2 [file CPR-52-e12598-s002.TIF]

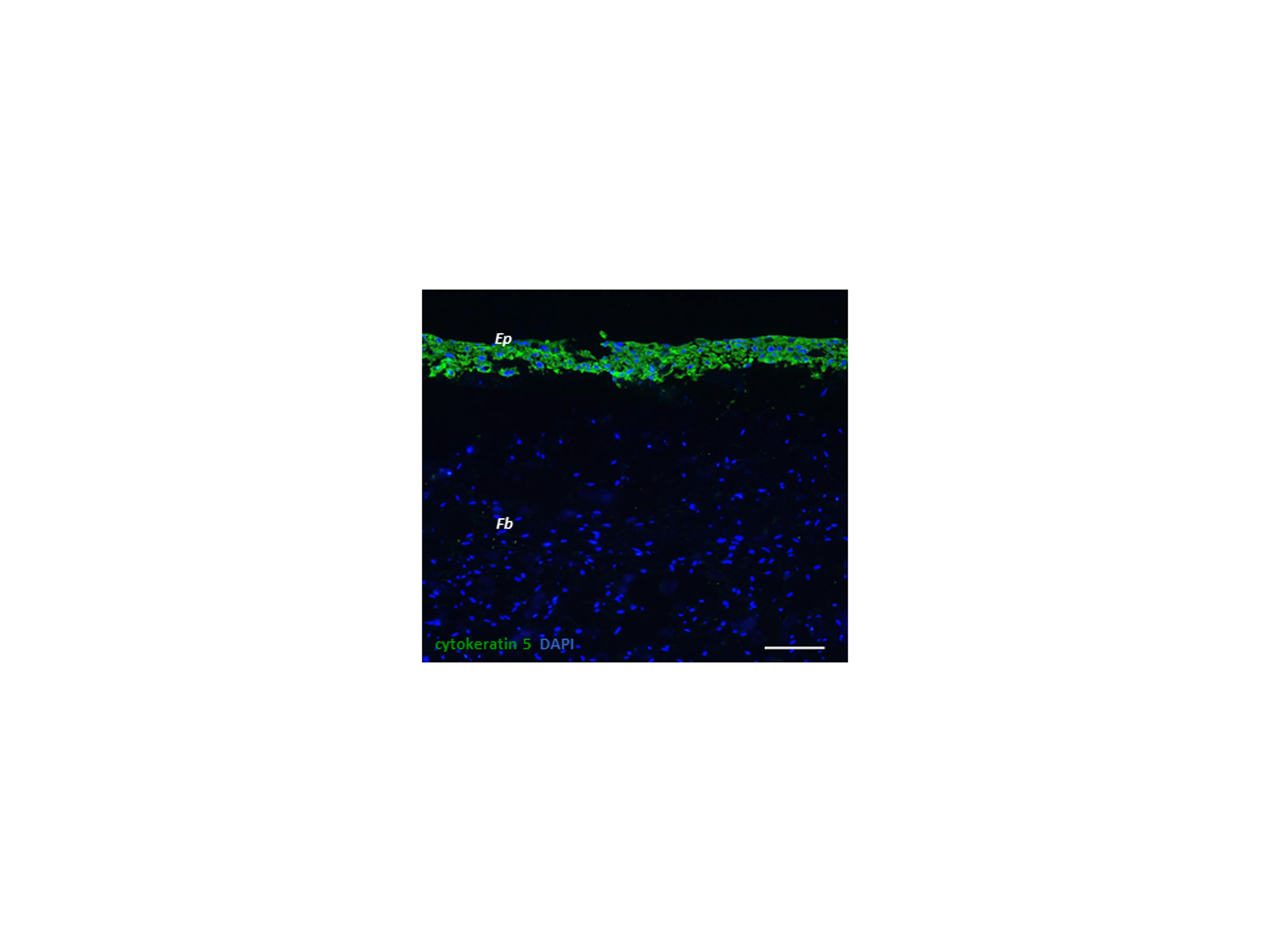

Supplement: Supplementary file 3 [file CPR-52-e12598-s003.tif]

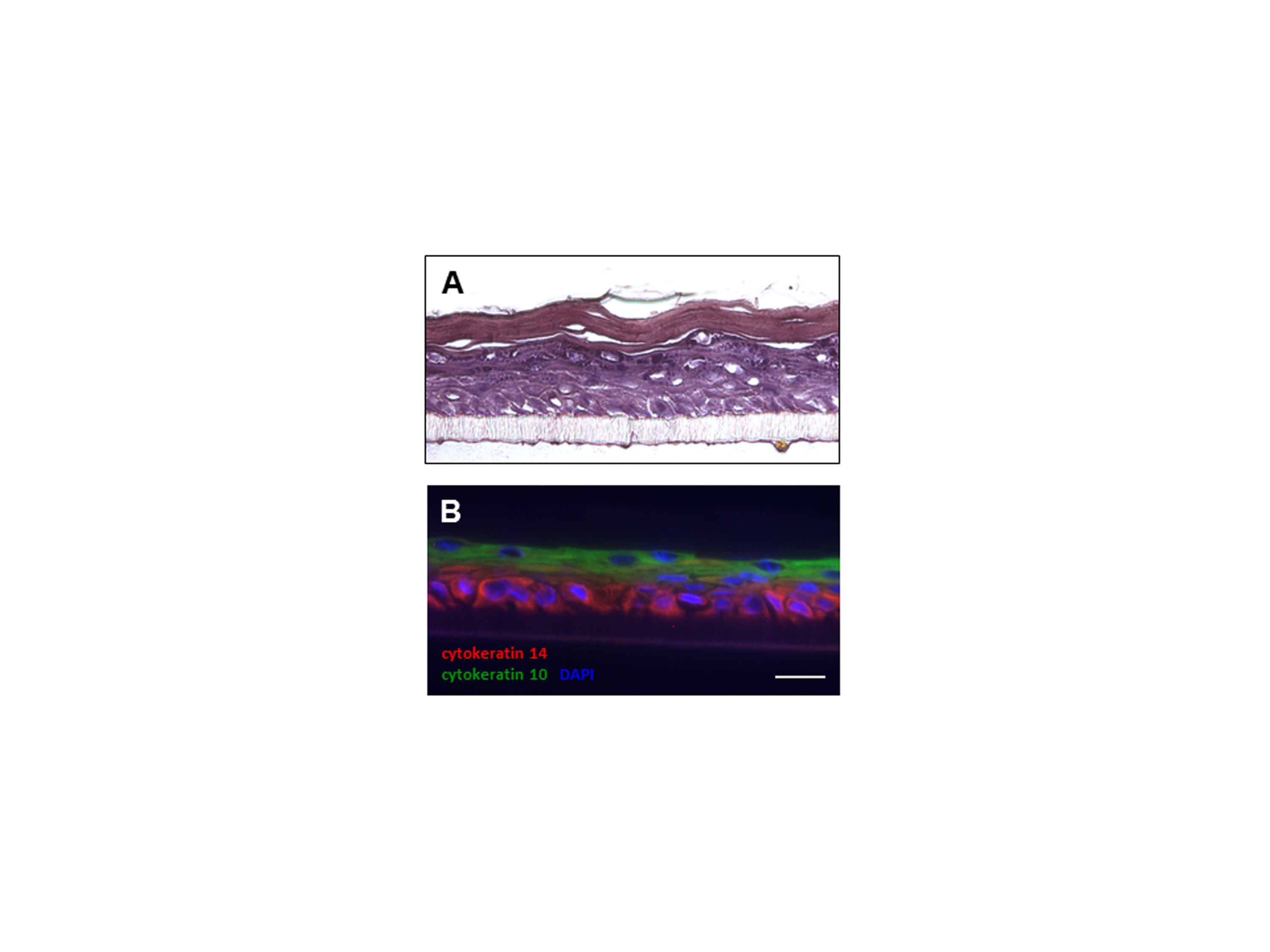

Supplement: Supplementary file 4 [file CPR-52-e12598-s004.tif]
