# Supplementary material for: Non‐woven bilayered biodegradable chitosan‐gelatin‐polylactide scaffold for bioengineering of tracheal epithelium
Source: Cell Prolif. 2019 Mar 21;52(3):e12598. doi: 10.1111/cpr.12598 (PMC6536443; doi:10.1111/cpr.12598)
Supplement: Supplementary file 7 [file CPR-52-e12598-s007.docx]

**Supporting information**

***Microfibrous layer: seeding with fibroblasts and change of mechanical properties during cultivation***

The microfibrous layer not only provides a mechanical support for a thin top nanofibrous layer, but also accommodates mesenchymal cells - fibroblasts. These cells possess a strong matrix-modifying ability (Grinnell et al., 2003) and hence mechanical properties of a microlayer may be a subject of significant changes during cell cultivation. As shown by our results, the strength of the cell-free nonwoven material decreased over time. The effects of fibroblasts presence appeared to be not prominent: moderate decline in material strength became noticeable at 14th day of cell cultivation **(Fig. S-2)**.

To compare stability of electrospun and lyophilized scaffolds, we assessed the changes in strength of nonwoven scaffolds and collagen sponges after cultivation with fibroblasts. Collagen sponges crosslinked with glutaraldehyde were used in this comparison given that this type of material is traditionally used for 3D cultivation of respiratory epithelium (Tada et al., 2008). Without cells, the strength of the acellular collagen sponge decreased only slightly after 14 days of incubation **(Fig. S-2 A)**. However, in the presence of fibroblasts, the strength of sponges dropped sharply to almost undetectable levels. By contrast, the electrospun CGP scaffolds seeded with fibroblasts retained the sufficient compressive modulus even after 30 days of incubation. The susceptibility of matrix to stretching (elongation) was also much more promoted by fibroblasts seeded on sponges as compared to electrospun material **(Fig. S-2 B)**. Thus, the nonwoven microfiber layer effectively supported primary fibroblast growth and retained sufficient mechanical properties during the process of cell cultivation. This allowed to obtain differentiated tissue equivalents, which require considerable time for formation and maturation *in vitro*. In perspective, this kind of the material could serve as a mucous substitute for restoring the barrier function in trachea. However, the mechanical strength of the scaffold is not sufficient to support inner tracheal lumen from external pressure. The rigidity of the trachea is mainly provided by cartilage rings.

1. Grinnell F. Fibroblast biology in three-dimensional collagen matrices. *Trends in cell biology*. 2003;13:264-9. *doi:* 10.1016/S0962-8924(03)00057-6
2. Tada Y, Suzuki T, Takezawa T, Nomoto Y, Kobayashi K, Nakamura T, Omori K. Regeneration of tracheal epithelium utilizing a novel bipotential collagen scaffold, *Ann Otol Rhinol Laryngol*. 2008;117:359-365. *doi:*10.1177/000348940811700506
